# Factors Affecting on Environmental Strategy in SMEs manufacturing Firms of a developing country: Mediating role of Green Employee Behavior

**DOI:** 10.12688/f1000research.152351.1

**Published:** 2024-06-18

**Authors:** Ikramuddin Junejo, Ummi Naiemah Saraih, Jorge Alberto Esponda Perez, Sarmad Ejaz, Faisal Ejaz, Md Billal Hossain

**Affiliations:** 1 Department of Managment Sciences, SZABIST University, Hyderabad, Pakistan; 2 Universiti Malaysia Perlis, Arau, Perlis, Malaysia; 3 Daffodil International University, Dhaka, Dhaka Division, Bangladesh; 4 Universiti Malaysia Perlis, Arau, Perlis, Malaysia; 5 Department of Management Sciences, Universidad de Ciencias y Artes de Chiapas, Tuxtla Gutiérrez, Chiapas, Mexico; 6 Department of Management Sciences, University of Okara, Okara, Pakistan; 7 School of international Relations, Minhaj University, Lahore, Pakistan; 8 Faculty of Economics, Széchenyi Istvàn University, Győr, Egyetem tér 1, 9026, Hungary

**Keywords:** Reduction in resource use; Greening of processes and products; Green employee behavior; Environmental strategy

## Abstract

**Background:**

Sustainability in small and medium-sized enterprises (SMEs) which engage heavily in manufacturing provides them with an essential platform for implementing environmental strategy, which is aimed at reducing environmental damage and promoting environmental protection. This study aims to examine the impact of the reduction in resource use, the greening of processes and products on environmental strategy and the mediating role of green employee behaviour.

**Methods:**

Primary data gathered with the help of questionnaires from employees of SMEs manufacturing in developing countries, Pakistan. The research gathered data from 211 participants by applying a pre-tested instrumental questionnaire and structural equation modelling (SEM) for data analysis.

**Results:**

The results confirmed all direct effects, including a reduction in resource use, the greening of processes and products and green employee behaviour of environmental strategies in small and medium manufacturing firms. Also, confirmed partial mediation effect of green employee behavior between greening of processes and products and environmental strategy.

**Conclusion:**

The current study clearly showed the need for programs and infrastructures that support resource reduction and greening businesses in SMEs. Companies’ implementation of green processes and technologies allows SMEs to take an environmentally responsible stand and become part of the movement toward sustainability. The research stresses the importance of green employee conduct as a mediator, the central point being the individual employee being engaged and aware of the subject and willing to participate in environmentally friendly practices. Therefore, small and medium enterprises should enlighten their staff to practice green behaviour, give them what they need and empower them to be change drivers that will trigger sustainability throughout the organisations.

## Introduction

All around the world, climate change, water pollution, air pollution and many other environmental issues are progressively becoming more serious, and they are already causing a great deal of anxiety concerning sustainability ( Manisalidis et al., 2020). They are so multifaceted that they create or sometimes extend the impacts on the ecosystem for human health and the balance of planet Earth. Calling for immediate, joint action from a person, a local community, and on governmental and business levels is necessary to address such environmental problems. Sustainable measures, which entail shifting to renewables, embracing eco-friendly technology, promoting recycling and waste reduction, adopting more stringent environmental regulation systems and promoting awareness about environmental conservation among people and other stakeholders, should be the focus of environmental sustainability. Governments, as well as regulatory bodies, also make contributions to the issue by encouraging social responsibility and environmental protection ( Camilleri, 2022). Several nations have enacted laws which compel companies to disclose their performance under particular sustainability criteria and have implemented financial incentives and tax rebates for businesses that subscribe to eco-friendly practices.

Nevertheless, it must be stated that deploying CSR and sustainability has diverse results among companies ( Sanchez-Planelles et al., 2022). There are instances when companies show so much greenwashing, meaning they claim to be eco-friendly or responsible in the social context without tangible modifications. Hence, all stakeholders ought to remain vigilant and employ the principle of critical thinking in evaluating whether or not the company has demonstrated its commitment to CRS and sustainability. The rise of CSR and the development of sustainability applications by enterprises is becoming the reflection of a quickly increasing attention towards safeguarding the natural environment while also keeping the economy growing ( Le et al., 2024). Through sustainability, businesses accrue the benefits of a chance to be part of structuring a sustainable world.

Sustainability in small and medium-sized enterprises (SMEs) which engage heavily in manufacturing provides them with an essential platform for implementing environmental strategy, which is aimed at reducing environmental damage and promoting environmental protection. Undertaking an environmental appraisal enables SMEs to recognize the high environmental impacts and areas of improvement ( Johnstone, 2021). This assessment can go into selecting power usage, waste creation, water utilization, and pollution. It makes the base for installing environmental targets and goals. Include employees in environmental business ideas and train them about sustainable methods. Make an environment for staff members to recommend developing our environmental activities and reward their efforts. Develop an environmental consciousness culture that is embedded within the organisations. Small- and medium-sized enterprises should consider the obstacles and limits they may face while developing environmental strategies, like small budgets or lack of experience ( Chien et al., 2021). Partnering with green manufacturing specialists, implementing applicable incentives by the government, and connecting the industry with the same brand are the keys to overcoming environmental sustainability challenges in the manufacturing industry.

A green way of life is any manner or activity that is advantageous to the environment or minimizes the impact on the environment ( D’Angelo et al., 2023). It means to be mindful of our actions, to make choices, and to take actions that assist in sustaining the environment and protecting natural resources. Green behaviour empowers individuals, social groups, public organisations and governments to adopt various practices from every point of life to make it environmentally friendly. Participating in activities that make people understand environmental problems, lending support to environment conservation organisations and advocating policies and ways of doing things that will sustain our environment (( Junejo et al., 2020; Shah et al., 2021; Zikargae et al., 2022). The constant accumulation of information about environmental issues, the constant access to the current best practices and innovations, and a constant urge to share the discovered information with others ensure the substantial progress of environmental awareness and green behaviour. The availability of practices for using materials, including paper, plastic, glass, and metal, would otherwise be destined for landfills. It saved waste generation by green consumption, upcycling pieces, composting organic waste and buying stuff in zero packaging packers.

Most earlier studies only focused on the factors of an individual’s either internal aspects or external influences, not combine to know how these elements interact with each other in context of environmental strategy ( Bertassini et al., 2021; Manninen & Huiskonen, 2022; Junejo et al., 2022). Nevertheless, some firms do not think just about the environment. However, they also see it as a social responsibility and do the best they can by cutting down on resource use and emissions. At the same time, they are aware that their actions may affect other people in society. Therefore, in this study, economic factors, such as resource savings and emissions, to operationalize environmental policy are taken as the context within the study. Until now, only the studies examining the impact of renewable energy have been carried out in the developed countries ( Eisenmenger et al., 2020; Ding et al., 2021; Naveed et al., 2023; Sohu et al., 2023). Hence, to narrow down the geographical area and because of the limitations of the period and the cost, this cross-sectional research will be conducted in developing country Pakistan and manufacturing companies.

## Literature review

### Theoretical foundation

According to the RBV, the resource-based view recommends that a company's performance and effectiveness stem from its unique resources and abilities ( Dionysus & Arifin, 2020). By this study, RBV is appropriate to explain that reducing natural resource use and making processes and products greener (independent variables) can help develop a sustainable competitive advantage through environmental strategy among (dependent variable), through environmental behaviour of employees acting as a mediating variable. It is circulated that RBV resources are subdivided into tangible and intangible resources according to the viewpoint of the resources-based view theory. This means that in this given case, the reduction of resource use would entail shrinking resource use by the factor of the primary tangible resources comprising energy, water, and raw materials. Development and implementation of eco-friendly technologies and procedures during all the other activities of the concern, which are featured as non-material assets of a company.

From the viewpoint of the RBV, eco-friendly firms have a golden chance to capture a more significant market share by using their resources in a manner that would communicate their green efforts both internally and to the outside world, leading to various benefits like reputation, efficiency, and satisfaction of the customers' demand for sustainable goods and services. Those factors can be a basis for customer centricity, cost efficiency and financial success. While the RBV insists that it implies more than just being a rich brand, it should be a rich brand where the resources are managed well and leveraged. Mediation plays a role in green employee actions here. The organization-friendly behaviour of green individuals implies that employees' internal personal activities and attitudes will be positively connected to the company's environmental sustainability initiatives ( Yang & Liu, 2022). This behaviour can include saving energy, decreasing waste, and becoming proactive in activities that promote sustainability.

### Hypothesis development

***Reduction of resource use***

Organizations could secure resources by saving them. As a result, companies will be able to apply their resources most efficiently ( Shkarlet et al., 2020). Such contribution will ensure emissions decline, reduced waste generation, and reduced pollution rates, all prerequisites for excellent environmental performance. Most reduction strategies in the resource field have places where the company wages are decreased ( Azizi et al., 2021; Bilal et al., 2024). For instance, installing energy-saving devices results in lower energy expenses, using waste-preventive measures reduces dumping expenses, and saving aluminum leads to input savings. Through these capital savings, the solidification of the business case for resource management is strengthened alongside environmental management improvement ( Afum et al., 2021; Sohu et al., 2024; Kherazi et al., 2024). These efforts can be the impetus for innovative technologies, new styles of work, and resource-efficient products. This benefit is that businesses can distinguish themselves from their competitors by being an environmentally conscious company that meets the evolving market needs for green products and services and positions itself as an esteemed environmental steward ( Kumari, 2024; Akhtar et al., 2023). Thus, the following alternative hypothesis is suggested.

*H1: Reduction of resource use is positively related to environmental performance.*

***Greening of processes and products***

There is a name known as eco-efficiency, which is the capacity that organizations have to provide goods and business processes that meet customer needs but generate a small amount of environmental impact ( Güngör et al., 2023; Hongyun et al., 2023). Furthermore, it highlights the alignment of environmental appraisal to the planning of production, design, and supply to balance sustainable economic development and environmental protection. A phase of reducing hazardous substances or pollutants always accompanies the phase where processes and products are greened. In addition to this, businesses can achieve this by applying cleaner production techniques, replacing harmful materials with safer alternatives, and adding pollution prevention techniques to minimize their impact on air, water, and soil quality ( de Mello Santos et al., 2022; Sohu, Hongyun, et al., 2020; Sohu et al., 2019). As a result, companies will benefit from improved environmental performance. Eco-designing goods means creating products with some green characteristics and making them efficient in their energy consumption, recycling, or overall environmental footprint. By providing eco-friendly alternatives to the market, corporations can cater to consumers' demand for environmentally friendly goods, build their sustainable brand reputations, and contribute to attaining optimum environmental performance ( Reddy et al., 2023; Mirani et al., 2021; Dakhan et al., 2021). Thus, the following alternative hypothesis is suggested.

*H2: Greening of processes and products is positively related to environmental performance.*

*H3: Greening of processes and products is positively related to green employee behavior.*

### Mediating role green employee behavior

The Social Cognitive Theory states that these are not the only ones that affect an individual's behaviour, but also personal factors, environment and cognitions ( Schunk & DiBenedetto, 2020; Sohu et al., 2022). Thus, according to the green self-crafting theory, employees' attitude, beliefs, and confidence in environmental sustainability issues might influence their organisational behaviour. When employees become involved or act actively on the green side, such as saving some resources, reducing waste, and promoting environmental sustainability, such acts' contributions help improve environmental performance ( Dakhan et al., 2020; Malik et al., 2021; Iqbal et al., 2023). The green behaviour of the employee is formed by the societal standards in the company, which define what is considered correct and acceptable. When peers watch other employees at work implementing resource reduction and environmentally friendly practices, a normative expectation develops that people should behave the same way to be part of this group ( Zacher et al., 2023; Sohu et al., 2020). Enabling everyone to see the good results caused by being sustainability-oriented and peer support among employees can create a work environment where most employees will start to adopt green behaviour.

In most cases, an employee's environmental sustainability behaviour reflects on dual aspects, which are willingness to learn and improve ( Ababneh, 2021). Using the systems of feedback, performance evaluation, and reward for sustainability there, worldwide establishments may prompt workers to improve their behaviour towards nature by looking for better ways to preserve the environment. This never-ending goal to produce endless improvements may also play an essential part in environmental performance by discovering new ways of reducing the consumption of natural resources or maintaining sustainability ( Chien et al., 2023). Thus, following alternative hypothesis is suggested.

*H4: Green employee behavior is positively related to green employee behavior.*

*H5: Green employee behavior mediates between reduction of resource use and environmental performance.*

## Methods

### Population, sample size and procedure

The data collection was done through cross-sectional research. This design allowed us to investigate relationships between employee behaviour in pursuing environmentally friendly strategies and reducing the use of resources and waste. The role of the researcher in primary data collection entails designing the data collection process, for example, by defining the template for a questionnaire as well as choosing the sample size and number of samples. Next, the researcher is directly involved in data gathering from the participants or sources. The current research analysis is based on primary data, which involves the top management employees of manufacturing companies in Sindh, Pakistan. The sample size of 211 participants was selected using a non-probability, convenience, and snowball sampling approach. Initial appointments were set up over phone calls, and the participants were instructed to be available for the print out questionnaire during the time frame. Our research procedure was as ethical as possible and carried out according to the principles and guidelines. The participants were informed before and provided with the opportunity to give consent.

Moreover, their private information was secure and anonymous. The study results were reported as a group, with the addition that no identifying information exists on individual participants. The structured questionnaire was framed and approved to retrieve information from the participants. The survey consisted of components designed to measure reduction in resource use, greening of processes and products, green employee behavior and environmental strategy.

### Ethics and consent

This study “ **Factors Affecting on Environmental Strategy in SME Manufacturing Firms of a Developing Country: Mediating Role of Green Employee Behavior”** was approved by the Ethics Committee of The University of Okara constitutes the departmental Ethics Approval Committee (REBSSH/2024/4-9) in January 03, 2024. All of the informants gave written and oral informed consent to participate in the study. Under the Personal Data Protection Act (PDPA) 2010, the researchers assure that all information collected is kept confidential and merely for the academic purposes. Therefore, they were assured that their responses would be kept in strict anonymity and reported as aggregate results.

### Measurements

Independent variable reduction in resources use four research items were adopted from the research of ( Čater et al., 2023). Another independent variable greening of processes and products also four items were taken from the study of ( Čater et al., 2023). In addition to this, mediating variable green employee behavior four items were taken from the study of ( Mi et al., 2020). Lastly, dependent variable Environmental strategy’s four research items adopted from the research of ( Čater et al., 2023).

### Statistical tool

SEM was used to test the hypotheses based on the data that we obtained from the survey. For the analysis of the data, version SEM 3 was leveraged. Structural equation modelling (SEM), a statistical method for studying highly complicated connections between multiple variables, allows for the analysis of such relationships ( Hair et al., 2021). SEM covers testing via confirmatory and exploratory factor analysis and employs path analysis to run tests of hypotheses that deal with a correlation of variables. According to SEM, researchers develop a model of latent and observed variables, which is compared with how the data behaves in the real world.

## Results and Discussion

### Reliability and validity (questionnaire)

Data integrity and validity are essential when they are known to be accurate and used as a tool for testing hypotheses ( Orsini et al., 2020). As it is observed that with excellent and decisive data, it is possible to obtain valid and authentic results of hypothesis testing, which can lead to some false conclusions. Hence, researchers should, as a matter of priority, begin with identifying and establishing credibility and authenticity in a study methodology before conducting a hypothesis ( Hancock et al., 2021). The research procedure is the foundation that determines the credibility of the study. This is why reliability and validity are considerations that need to be implemented before any hypothesis testing is done ( Sürücü & Maslakci, 2020). The reliability deals with the continualness and stability of the data collection tool or method used at the time. Reliability represents the degree to which the measurement tool or method used to collect data correctly portrays reality.

**Figure 1.**
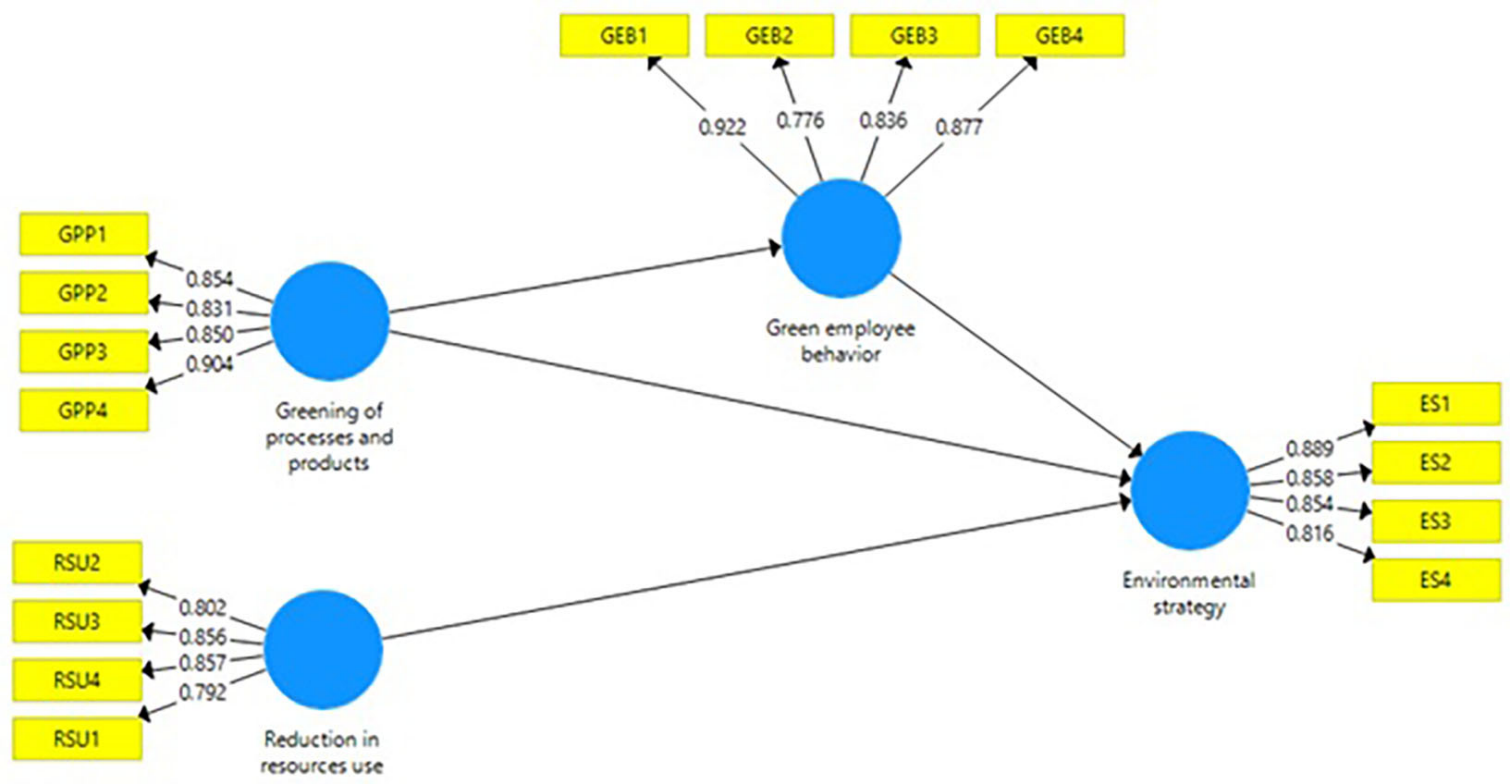
Measurement model. Source: Authors’ own estimations.

There are two ways to appraise a study's reliability, one of which is the measurement of Cronbach's alpha, and the other is the utilization of Composite Reliability ( Hayes & Coutts, 2020). The researchers favor reporting both of these indices if they are available. Nonetheless, it substantiates the point that reliability is the fundamental part of data quality apart from establishing the validity and accuracy of the instrument or method before the researchers start to make a hypothesis. A Cronbach's Alpha and Composite Reliability of 0.70 and above is usually considered acceptable, although higher values are to be advocated for more excellent reliability. Moreover, all the values for the current are more than 0.70. Cronbach's alpha and Composite Reliability values are given in Table 1 and Figure 1.

**Table 1.** Reliability and validity (questionnaire).

Factors	Item SPSS coding	Items loading	Cronbach alpha value	Composite reliability	Average Variance Extraction (AVE)
**Reduction in resources use**	RSU1	0.792	0.846	0.896	0.684
	RSU2	0.802			
	RSU3	0.856			
	RSU4	0.857			
**Greening of processes and products**	GPP1	0.854	0.883	0.919	0.740
	GPP2	0.831			
	GPP3	0.850			
	GPP4	0.904			
**Green employee behavior**	GEB1	0.922	0.876	0.915	0.730
	GEB2	0.776			
	GEB3	0.836			
	GEB4	0.877			
**Environmental strategy**	ES1	0.889	0.877	0.916	0.731
	ES2	0.858			
	ES3	0.854			
	ES4	0.816			

Source: Authors’ own estimations.

Converged A average variance extracted (AVE) is a widely used validity measure of construct validity in SEM models. A CFI value greater than 0.50 is usually deemed acceptable for the construct validity, though this will vary based on the type of research being conducted and the number of items on a scale. They have a positive value for the present day, with all current values being more significant than 0.50. Average Variance Extracted values are given in Table 1 and Figure 1.

**Figure 2.**
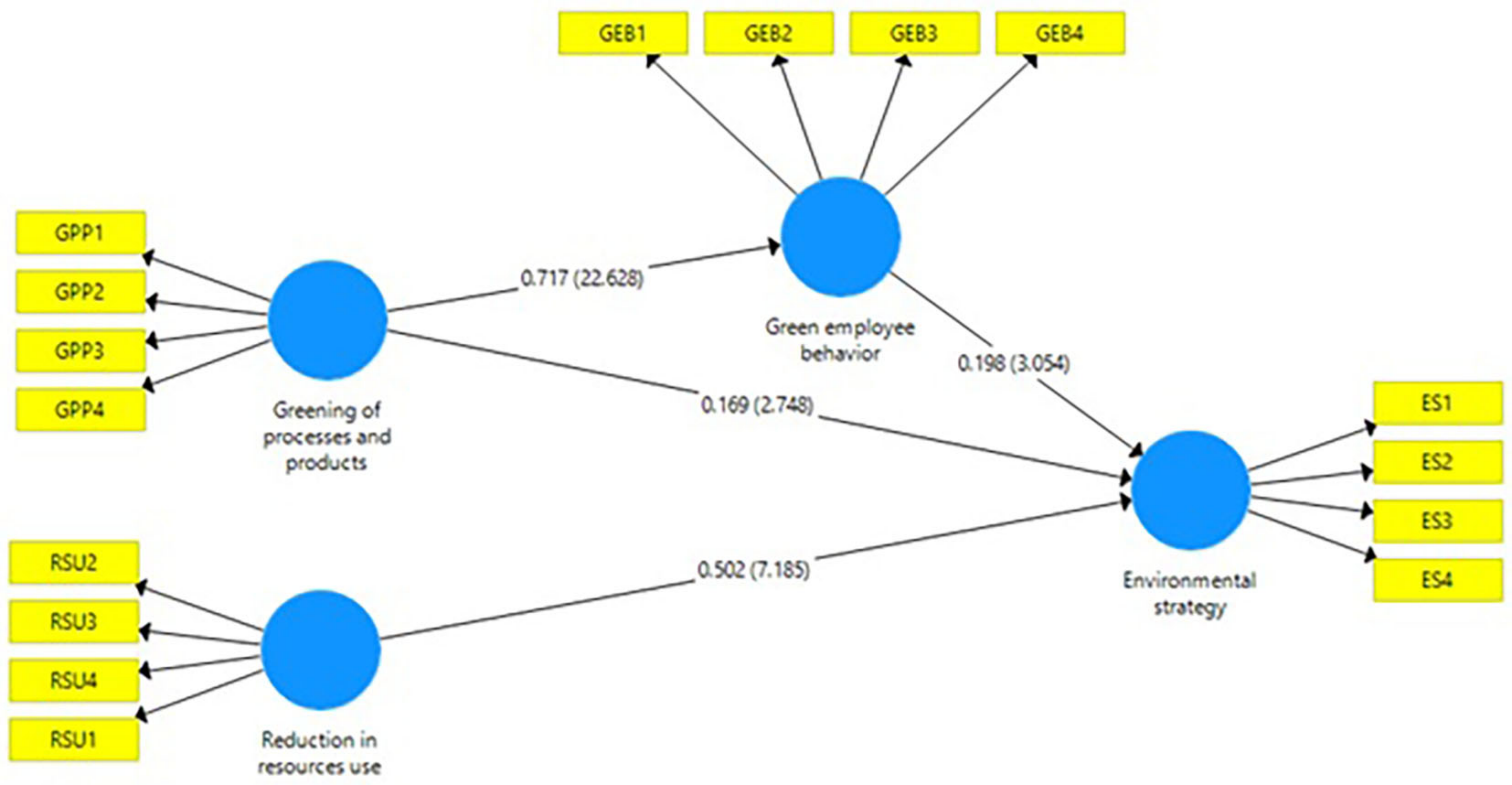
Structural model. Source: Authors’ own estimations.

### Hypotheses testing and discussion

*H1:* The hypothesis of reduction in resource use and environmental strategy. This beta value of 0.502 reflects a positive and significant association on environmental strategy. The t-value of 7.429 is also significant. Hence, hypothesis is accepted and null is rejected (see Table 2 & Figure 2). Many researchers have indicated that saving some resources is now a primary catalyst or momentum as most organizations adopt environmental programs ( Sarja et al., 2021). By their dedication to controlling resource consumption, their actions signify the pledge to protect the environment and sustainability. This dedication is illustrated by several implementation strategies, including energy-saving technologies, the optimization of production to eliminate waste and sustainable purchase methods ( Hegab et al., 2023). Organizations that minimize resource exportation will have a lower impact on the environment and help them cut costs and become more efficient and effective in the long term. Research evidence suggests that companies keen on the conservation of resources might embed a complete sustainability strategy in that category.

*H2:* This hypothesis states that green processes and products and environmental strategy. Accordingly, to beta value 0.169, there is a positive association with *H1.* In addition to this, the value of t = 2.834 is a statistically significant which means green processes and products has positive and significant impact on environmental strategy (see Table 2 & Figure 2). The study result showed that the pro-environment strategy derived from the decision-makers who choose green processes and products is consistent with the earlier conclusion on how organizations can practice sustainability ( Xin et al., 2023). In pursuing green processes and products, companies seek to decrease their environmental footprint, cut emissions, and become more environmentally friendly. Such could be through embracing clean technologies, using renewable energy sources, and implementing eco-conscious design ( Sengupta & Hussain, 2024).

*H3:* impact of green employee behavior on environmental strategy. The estimation of the beta value as 0.717 means that there is a significant positive correlation between these variables. The t-value of 22.325 strongly shows a significant relationship with these variables (see Table 2 & Figure 2). The research results back up the arguments that a robust and direct link exists between the greening of processes and products and workplace activity. Organizations that sustain and integrate sustainability will discover environmentally positive employee engagement ( Raza et al., 2021). This is a similar result to the previous research that points out the organizational practices and policies that serve how employees conceive and act towards sustainability issues. Promoting an environmental-responsibility culture among organizations could improve their environmental performance and achieve sustainable societal objectives ( Channa et al., 2021).

*H4:* The hypothesis proposes that green employee behavior on an environmental strategy. Beta 0.198 encourages a positive relationship. The t statistic of 3.055 is also suggested a significant role of green employee behavior and environmental strategy (see Table 2 & Figure 2). It could imply a meaningful relationship. The discovery of a positive association between eco-friendly employee activities and the consideration of the green strategy within business contexts complements the assumption that the employee's participation is one of the leading factors that foster the implementation of meaningful sustainability programs in companies. This result is proof of the fact that the previous studies have brought out the role of employee conduct in the organizing of sustainability activities and the development of sustainability policies ( Al-Swidi et al., 2021). Organizations can step up to the challenge of environmental awareness by creating an “eco-friendly employee” culture. This will make their contribution to protecting the environment and broader sustainability goals more significant.

H5: This study's hypothesis concerns the mediating role of green employee behavior between green of processes and products 0.142, the beta value and t-value=2.991, indicates a positive and significant relation (see Table 2 & Figure 2). The positive connection between greener products and employee behaviour with adopting an environmental strategy, which aligns with the approach to sustainable actions that should use all means from organizational practices and individual behaviours, supports the assumption that combining the positive inputs gives the best result. Organizations attempting to do both develop green processes and products while encouraging green employee behaviour, create a ripple effect which can, in turn, be used to drive holistic environmental policies ( Fernandes et al., 2021). The studies that were conducted agree with the previous research; they affirm that both organizational mechanisms and employee activities have a role to play in the sustainability objectives ( Lu et al., 2023; Ren et al., 2022). By incorporating green activities and eco-awareness for employees, organizations' environmental actions can strengthen, improving their sustainability achievements.

**Table 2.** Path directions.

Paths	Value of beta	Standard Deviation	T-Value	Remarks
Reduction in resources use -> Environmental strategy	0.502	0.068	7.429	Accepted
Greening of processes and products -> Environmental strategy	0.169	0.060	2.834	Accepted
Greening of processes and products -> Green employee behavior	0.717	0.032	22.325	Accepted
Green employee behavior -> Environmental strategy	0.198	0.065	3.055	Accepted
Greening of processes and products -> Green employee behavior -> Environmental strategy	0.142	0.047	2.991	Accepted

Source: Authors’ own estimations.

## Concluding remarks

The outcomes showed that organizations can perform the best in the environment by putting green products, processes, technologies, and environment-focused action plans into tactics. Resource minimization is a determinant of successful environmental performance since it encourages improved resource efficiency, decreased emissions, and reduced volume of waste. Adopting eco-friendly practices not only helps optimize the performance of ecological processes and products but also entails employing environmentally friendly materials and technologies throughout the product’s life cycle. Green employee behaviour represents an essential mediator on both ends of the equation, giving life to methodologies applied in resource-saving and implementation of green practices, which might result in saving the planet from environmental degradation. This contributes to a culture where the desired change to sustainability, innovation, and continuous improvement occurs. Also, environmental improvement has quite reasonable chances to be accomplished by organizations whose environmental schemes have been well-designed and implemented in all departments. An environmental strategy is a critical point of how the organization makes promises to be environment friendly and sets the objectives, integrating environmental factors into the business activities.

### Practical implication

Organizations should give forests the top value in the durability of resources during their operations. It may encompass using energy-saving technologies, taking an in-depth look into production processes, reducing water consumption, and reducing waste production. Emphasizing the streamlining approach in these institutions can improve cost-efficiency, lower costs, and minimize environmental influence. Therefore, businesses should not wait but rather play leading roles by embracing eco-friendly practices and technologies to green their processes and products. This comprises, among others, the adoption of renewable energy, green production processes, recycling, waste reduction, and sustainable materials. By being environmentally friendly and adopting green technologies, businesses can minimize risks to the ecosystem, profile sustainability, and meet the eco-conscious consumer's requirement for green products and services. Organizations should develop an all-inclusive environmental policy with specific goals and an outline of the achieved aims, measures, and policies. This activity stream should fully agree with the company's general strategic map and should align with the functional areas. Moreover, for the company to have a clearly defined environmental policy, it provides a pathway for sustainable actions, communicating accurately, and designing a system that helps to develop the organization's environmental performance.

### Limitations and future research directions

One possible limitation of such research is that it is one where manufacturing companies are the key players, potentially limiting the approachability of its outcomes to other categories or sectors. Further, it does not give details on how these environmental practices' side effects or price balancing could work together to identify the best cases to achieve sustainability in manufacturing companies. The role of mediation can be observed in the times to come via measures like environmental education and environmental approval, etc., in a developing country like Pakistan. Eventually, this research should be confirmed and expanded to verify the more precise causes and to explore the broader contextual factors that play a role in environmentally friendly practices in different industries and sectors.

**Availability of data and material**: is available with the manuscript.

#### Author contribution

Authors equally contributed to the analysis and writing of this publication.

#### Ethics and consent

This study **“Factors Affecting on Environmental Strategy in SME Manufacturing Firms of a Developing Country: Mediating Role of Green Employee Behavior”** was approved by the Ethics Committee of The University of Okara constitutes the departmental Ethics Approval Committee (REBSSH/2024/4-9) in January 03, 2024. All of the informants gave written and oral informed consent to participate in the study. Under the Personal Data Protection Act (PDPA) 2010, the researchers assure that all information collected is kept confidential and merely for the academic purposes. Therefore, they were assured that their responses would be kept in strict anonymity and reported as aggregate results.

## Data Availability

Figshare: Dataset & Questionnaire: **DOI:** https://doi.org/10.6084/m9.figshare.25975462.v1 This project contains the following underlying data:
• Data set: Data set.csv • Questionnaire: Environmental Strategy Questionnaire.xlsx This project contains the following underlying data: **Figshare:** Figure 1_Measurment model.docx **Figshare:** Figure 2_Stutrual model.docx **DOI:** https://doi.org/10.6084/m9.figshare.25975387.v1 This project contains the following underlying data: **Figshare:** Table 1_Measurment model **Figshare:** Table 2_Structural model **DOI:** https://doi.org/10.6084/m9.figshare.25975438.v1 Data are available under the terms of the Creative Commons Zero “No rights reserved” Attribution 4.0 International (CC BY 4.0)
