# Supplementary material for: Factors Affecting Environmental Strategy in SME Manufacturing Firms of a Developing Country: Mediating Role of Green Employee Behavior
Source: F1000Res. 2025 Jul 7;13:654. Originally published 2024 Jun 18. [Version 2] doi: 10.12688/f1000research.152351.2 (PMC11364969; doi:10.12688/f1000research.152351.2)
Supplement: Peer review history

# Reviewer report: Reviewer response for version 2

**DOI:** 10.5256/f1000research.180705.r397257

**Published:** 2025-08-20

**Authors:** Chun-Hung Cheng

**Affiliations:** 1 National Formosa University, Huwei, Taiwan Province, Taiwan

- The references cited in this paper are all from the past five years and are of high credibility.
- The proposed conceptual framework is clearly structured.
- The overall research design is sound and well-executed.
- It is recommended that the conclusion include more practical implications to serve as a reference for industry, thereby further highlighting the value of the research.

Is the work clearly and accurately presented and does it cite the current literature?

Yes

If applicable, is the statistical analysis and its interpretation appropriate?

Yes

Are all the source data underlying the results available to ensure full reproducibility?

Yes

Is the study design appropriate and is the work technically sound?

Yes

Are the conclusions drawn adequately supported by the results?

Partly

Are sufficient details of methods and analysis provided to allow replication by others?

Yes

Reviewer Expertise:

My research field focuses on the study of green operations and the implementation of green production-related tools in small and medium-sized enterprises (SMEs).

I confirm that I have read this submission and believe that I have an appropriate level of expertise to confirm that it is of an acceptable scientific standard.

# Reviewer report: Reviewer response for version 1

**DOI:** 10.5256/f1000research.167100.r315835

**Published:** 2024-09-03

**Authors:** Chun-Hung Cheng

**Affiliations:** 1 National Formosa University, Huwei, Taiwan Province, Taiwan

The literature review in this theoretical foundation can support the structure of the entire article's investigation. It is recommended that this article should supplement the research objectives. If the article only explains through hypotheses, it merely verifies the theory of the article. It would be better to clearly state the research objectives of this article and whether the conclusions drawn from the investigation align with these research objectives. Additionally, it is suggested that the article should supplement the background by explaining the current situation of the surveyed populations' country. This will further highlight the contribution of the article's conclusions.

Is the work clearly and accurately presented and does it cite the current literature?

Yes

If applicable, is the statistical analysis and its interpretation appropriate?

Yes

Are all the source data underlying the results available to ensure full reproducibility?

Yes

Is the study design appropriate and is the work technically sound?

Yes

Are the conclusions drawn adequately supported by the results?

Partly

Are sufficient details of methods and analysis provided to allow replication by others?

Yes

Reviewer Expertise:

My research field focuses on the study of green operations and the implementation of green production-related tools in small and medium-sized enterprises (SMEs).

I confirm that I have read this submission and believe that I have an appropriate level of expertise to confirm that it is of an acceptable scientific standard, however I have significant reservations, as outlined above.

# Reviewer report: Reviewer response for version 1

**DOI:** 10.5256/f1000research.167100.r315831

**Published:** 2024-08-30

**Authors:** Jan Muhammad Sohu

**Affiliations:** 1 Jiangsu University, Zhenjiang, Jiangsu, China

Thank you for the opportunity to review the paper titled "Factors affecting on environmental strategy in SMEs manufacturing firms of a developing country: mediating role of green employee behavior". Overall, the paper is well-written and the topic is interesting and well-justified.

**Minor revisions are suggested as follows:**

1. In the Abstract, the method section should mention the software tool(s) used, such as the SmartPLS version.
2. In the keywords, "SMEs" and "developing country" should be added to the list of keywords.
3. In the Introduction section, the research objectives should be clearly stated before the research contribution of the present study.
4. In the Conclusion section, the theoretical contribution of the study is missing and should be added.

Overall, the paper is of good quality, and with the minor revisions suggested, it should be ready for indexing. I wish the authors all the best.

Is the work clearly and accurately presented and does it cite the current literature?

Yes

If applicable, is the statistical analysis and its interpretation appropriate?

Yes

Are all the source data underlying the results available to ensure full reproducibility?

Yes

Is the study design appropriate and is the work technically sound?

Yes

Are the conclusions drawn adequately supported by the results?

Partly

Are sufficient details of methods and analysis provided to allow replication by others?

Partly

Reviewer Expertise:

e.g. Digital Marketing, Business Performance, and development, Big Data Analytics Capability, Digital Trasformation, Digital Innovation, Value Co-creation, and Green Supply Chain Management Practices.

I confirm that I have read this submission and believe that I have an appropriate level of expertise to confirm that it is of an acceptable scientific standard.

# Reviewer report: Reviewer response for version 1

**DOI:** 10.5256/f1000research.167100.r302073

**Published:** 2024-08-07

**Authors:** Andrew Kweku Conduah

**Affiliations:** 1 University of Professional Studies, Madina, Ghana; 2 University of Professional Studies, Legon, Legon, Ghana

The paper thoroughly examines environmental strategies within SMEs in a developing country, utilizing the Resource-Based View (RBV) to frame its analysis. The introduction effectively sets the stage by highlighting global environmental challenges and the urgency of addressing these issues. It situates the discussion within a broader international context, referencing trends in sustainability such as renewable energy, eco-friendly technology, and recycling. However, the introduction could be enhanced by including specific global case studies, offering a richer context and illustrating the broader application of the study’s findings. Additionally, while the problem statement is relevant, it lacks specificity concerning the unique challenges SMEs face in developing countries like Pakistan. Clearly defining the problem statement and articulating the study’s objectives more explicitly would strengthen the research foundation and provide clearer direction for the study.

 In the literature review, the paper offers a comprehensive overview of the theoretical foundation, effectively utilizing the RBV to explain how reduced resource use and green processes contribute to a competitive advantage through environmental strategies mediated by green employee behaviour. This integration of theoretical perspectives with practical implications is a notable strength, emphasizing the role of both tangible and intangible resources in achieving ecological sustainability. Nonetheless, discussing how green employee behaviour mediates the relationship between resource use and environmental performance requires more depth. A more detailed exploration of the mechanisms by which green employee behaviour influences this relationship would enhance the reader's understanding. Additionally, each hypothesis should be more explicitly connected to the RBV and supported by empirical evidence.

 The study's strengths lie in its effective highlighting of global environmental issues and its connection of local contexts to global sustainability efforts through discussions on renewable energy, eco-friendly technology, and recycling. The application of the RBV is appropriate and well-articulated, integrating theoretical perspectives with practical implications. However, the paper would benefit from addressing certain weaknesses. For example, it lacks explicit comparisons to specific global case studies and needs a more detailed problem statement addressing SMEs' unique challenges in developing countries. The study’s objectives are implied but not clearly articulated, and the explanation of the mediation role of green employee behaviour needs further elaboration. Furthermore, the hypotheses could be more clearly linked to the theoretical framework.

 To improve the paper, it is recommended that the unique challenges faced by SMEs in developing countries such as Pakistan be specified and the problem statement be defined clearly. This should include a focus on specific environmental strategy challenges for SMEs. The study’s objectives should be articulated more distinctly, encompassing economic factors and the mediating role of green employee behaviour. A more detailed explanation of how green employee behaviour acts as a mediator is needed, including a discussion of specific actions and attitudes contributing to improved environmental performance. Additionally, the connection between the hypotheses and the theoretical foundation should be clarified and strengthened, ensuring that each hypothesis is explicitly grounded in the RBV and supported by relevant literature.

 The study's methodological approach is sound. It includes a cross-sectional design, ethical data collection procedures, validated measurement tools, and Structural Equation Modeling (SEM) to address the research questions. This methodology is appropriate and effectively supports the study’s objectives.

 In terms of analysis, the study demonstrates high reliability and validity. The measurement tools show strong Cronbach's Alpha and Composite Reliability values, indicating consistent and stable data collection methods. Convergent validity is confirmed by Average Variance Extracted (AVE) values that exceed the threshold of 0.50. The hypotheses tested reveal significant findings: reducing resource use (H1) is positively associated with robust environmental strategies; greening of processes and products (H2) enhances environmental strategies; and green employee behaviour (H3) significantly boosts environmental strategy. Additionally, green employee behaviour contributes positively to environmental strategies (H4) and mediates the effect of green processes/products on environmental strategy (H5).

 The study highlights that effective environmental performance in organizations is achieved through the strategic implementation of green products, processes, and technologies. Resource minimisation is crucial for enhancing efficiency, reducing emissions, and minimising waste. Integrating eco-friendly practices and materials throughout the product lifecycle is essential for optimizing environmental performance. Green employee behaviour is a significant mediator, facilitating the adoption of green practices and contributing to sustainability and innovation within organizations. A well-designed environmental strategy, embedded across all departments, ensures commitment to sustainability and integrates environmental considerations into business activities.

 Practically, organisations should prioritize resource durability by adopting energy-saving technologies, optimizing production processes, reducing water consumption, and minimizing waste. Embracing eco-friendly practices such as renewable energy use, green production, recycling, and sustainable materials enhances cost-efficiency and minimises environmental impact. Companies should develop comprehensive environmental policies with specific goals and measures aligned with their strategic plans. This approach fosters sustainability, meets eco-conscious consumer demands, and strengthens the company’s environmental profile.

 The study’s limitations include its focus on manufacturing companies, which may restrict the applicability of its findings to other sectors. It also does not address potential side effects or cost implications of implementing environmental practices. Future research should explore the role of mediation through environmental education and approval, especially in developing countries like Pakistan. Expanding the research to encompass diverse industries and sectors will help verify findings and identify broader contextual factors influencing environmentally friendly practices.

Is the work clearly and accurately presented and does it cite the current literature?

Yes

If applicable, is the statistical analysis and its interpretation appropriate?

Yes

Are all the source data underlying the results available to ensure full reproducibility?

Yes

Is the study design appropriate and is the work technically sound?

Yes

Are the conclusions drawn adequately supported by the results?

Yes

Are sufficient details of methods and analysis provided to allow replication by others?

Yes

Reviewer Expertise:

§  Population Estimates and Projections.§  Population Health and Mortality- Health Emergencies & Preparedness, Ageing & Intergeneration Relations, and Non-Communicable Disease & Health Projection (Physical Activity, Obesity, Tobacco Control, etc.)§  Business Demography§  Business Policy and Strategy§  Business Management§  Public Administration§  Entrepreneurial Innovation

I confirm that I have read this submission and believe that I have an appropriate level of expertise to confirm that it is of an acceptable scientific standard.
